# Valorization of flowers and their role in circular bioeconomy and sustainable development goals

**DOI:** 10.1186/s40643-025-00954-w

**Published:** 2025-10-25

**Authors:** Harsh Kumar, Shivani Guleria, Rajni Dhalaria, Neetika Kimta, Nidhi Sethi, Daljeet Singh Dhanjal, Talwinder Kaur, Manish Kumar, Hasnita Binti Che Harun, Ashima Mahajan, Tabarak Malik, Eugenie Nepovimova

**Affiliations:** 1 Centre of Advanced Technologies, Faculty of Science, University of Hradec Kralove, Rokitanskeho 62, 50003 Hradec Kralove, Czech Republic; 2 Departmen of Food Science and Technology, Graphic Era (Deemed to be University), Dehradun, Uttarakhand 248002 India; 3 Department of Biotechnology, TIFAC-Centre of Relevance and Excellence in Agro and Industrial Biotechnology (CORE), Thapar Institute of Engineering and Technology, Patiala, 147001 India; 4 Department of Biosciences, Minerva PG College of Arts, Science and Commerce, Indora, Kangra, Himachal Pradesh 176402 India; 5 School of Biological and Environmental Sciences, Shoolini University of Biotechnology and Management Sciences, Solan, 173229 India; 6 Department of Pharmaceutical Sciences, Guru Nanak Dev University, Amritsar, 143005 India; 7 School of Bioengineering and Biosciences, Lovely Professional University, Phagwara, Punjab 144411 India; 8 Department of Microbiology, Guru Nanak Dev University, Amritsar, 143005 India; 9 Department of Food Technology, Guru Jambheshwar University of Science and Technology, Hisar, 125001 India; 10 Department of Agriculture, Faculty of Agro Based Industry, Universiti Malaysia Kelantan, 17600 Jeli, Malaysia; 11 School of Agriculture, Abhilashi University, Mandi, Himachal Pradesh 175028 India; 12 Department of Biomedical Sciences, Institute of Health, Jimma University, Jimma, Ethiopia; 13 Division of Research and Development, Lovely Professional University, Phagwara, Punjab 144411 India; 14 Department of Chemistry, Faculty of Science, University of Hradec Kralove, 50003 Hradec Kralove, Czech Republic; 15 Centre for Advanced Innovation Technologies, VSB-Technical University of Ostrava, 70800 Ostrava-Poruba, Czech Republic

**Keywords:** Environment, Flowers, Functional food, Human health, Safety

## Abstract

Green biomass has always played a crucial role in fulfilling sustainable development goals (SDGs), be it in real or waste form. Simultaneously, these biomasses have also justified the circular bioeconomy concept by prioritizing the restoration and safeguarding of ecosystems, thus focusing on exploiting renewable biological resources along with the waste streams associated with them for producing value-added products. Edible or non-edible, flowers found in nature are best suited for biomass in this category. Primarily, the flowers have been considered as the source of fragrance, hence explored by the beauty and cosmetic industry only. This review highlights the harnessing of flowers in producing bio-based nanomaterials, along with the functional food’s enrichment, therefore emphasizing their nutritional and physiological advantages. The present analysis thoroughly corresponds with SDG2 (zero hunger), SDG3 (Good health and well-being), SDG6 (clean water and sanitation), SDG8 (decent work and economic growth), and 9 (industry innovation and infrastructure), as well as the circular bioeconomy idea. Besides this, the review also examines the safety considerations related to its utilization.

**Graphical abstract:**

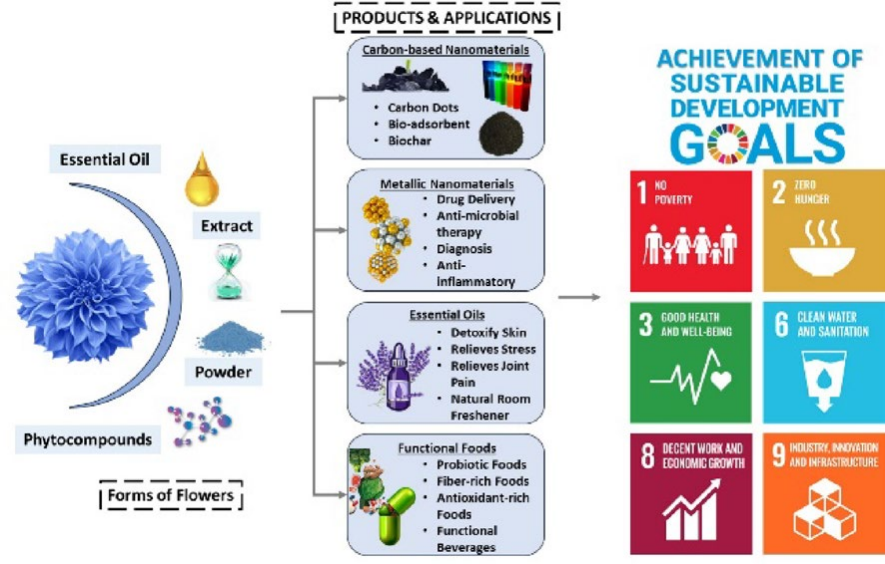

## Introduction

Consumption of flowers has been a common practice in ancient Greece and Rome as well as in ancient China as a constituent of traditional culinary or alternative medicines (Takahashi et al. 2020). According to the available sources, nearly 180 species of edible flowers have been used, spanning 100 genera and belonging to 97 plant families (Lu et al. 2016). The most widespread species used include roses (*Rosa* spp.), saffron (*Crocus sativus*), common marigold (*Calendula officinalis*), violet (*Viola odorata*), elder (*Sambucus nigra*), dandelion (*Taraxacum officinale*), chrysanthemum (*Chrysanthemum coronarium*), lilac (*Syringa vulgaris*), daylily (*Hemerocallis fulva*), mint (*Mentha* spp.), pansy (*Viola* x *wittrockiana*), tulip (*Tulipa* spp.) or nasturtium (*Tropaeolum majus*) (Fernandes et al. 2017; Mlcek and Rop 2011). Given this diversity, it is very important to identify the edible flowers properly. Edible flowers are commonly characterized as benign, nontoxic flowers that provide health advantages and are used by people in their diet (Acikgoz 2017). Since ancient times, dried chamomile (*Chamomilla recutita* L.) flowers have been used to cure a variety of illnesses, including menstrual disorders, sleeplessness, ulcers, haemorrhoids, and more (Srivastava et al. 2010). This is a prime example of the traditional use of medicinal flowers. In a similar vein, folk medicine has long employed the flowers of *Achillea millefolium* L., *Arnica montana* L., *Bellis perennis* L., *Calendula officinalis* L., and *Chamaemelum nobile* L. for a variety of medicinal uses (Garcia et al. 2021). The religious ceremonies held in the regions of Asia include flowers and garlands that have ritualistic importance and are used to display devotion. Unfortunately, these flowers utilized in religious ceremonies are considered sacred and are therefore not disposed of in landfills (Gupta et al. 2024). During 2019–2024, a growth rate of 20.1% was estimated for the floriculture market, thus pointing towards a substantial increment in the floral waste numbers in India (Gupta et al. 2024).

The circular bioeconomy (CBE) concept represents a significant departure from standard waste recycling approaches. This paradigm stresses both recovery and the maintenance of the resilience of productive ecosystems (Muscat et al. 2021). According to Carus and Dammer (2018), “the concept of a circular bioeconomy involves utilizing renewable bio-resources and converting them, along with their by-products, into valuable commodities including food, bioenergy, and animal feed.” The CBE approach also generates newer job opportunities. The frameworks have achieved considerable attention owing to their potential in facilitating the Sustainable Development Goals (SDGs) achievements (Albrecht et al. 2018). The Millennium Development Goals’ shortcomings were fully addressed by the Sustainable Development Goals (SDGs), which were accepted in 2015 as the main framework of the UN 2030 Agenda for Sustainable Development (Ahrens et al. 2025). The SDGs, in contrast to their predecessors, place a strong focus on revolutionary shifts and encourage the integration of social, economic, and ecological aspects as a single framework for global development (Scharlemann et al. 2020). Agroforestry, a land-use technique that incorporates trees with crops and occasionally livestock, offers a lot of promise to promote sustainability in this regard. Agroforestry produces landscapes that fulfil several purposes by utilising the same land to provide clean water, food, and renewable energy while also preserving biodiversity. As demonstrated by its Land Equivalent Ratio (LER > 1), when properly managed with complementary mixtures of trees, crops, and animals, it improves resource efficiency and supports the idea of “sustainable intensification” (Montagnini and Metzel 2018; Colfer et al. 2015).

The review underlines the potential of the flower*s* in accomplishing multiple SDGs, viz., economic growth and decent work, sanitation and clean water, good health as well as well-being, industry, innovation and infrastructure, zero hunger and no poverty. The current study aims to investigate the utilization of flowers in producing green biomass with varied applications. As a result, with the CBE idea and to attain SDGs, an attempt has been made to develop varied goals for embracing the major applied domains. The primary goal of the study is to investigate the benefits of metallic nanoparticles and green carbon dots created from various floral biomass, stressing their wide range of applications in treatments (SDG 3, and 6) and water management. The second objective of the study is to highlight the development of bio-adsorbents and biochars, thereby underlining their impactful role in water bioremediation and construction sectors (SDG 6, and 9). Thirdly, this study aims to investigate the essential oil potential derived from flowers (SDG 8). The fourth goal is to investigate the successful integration of various edible floral biomasses for improving the quality of a variety of food products, such as cereal-based meals, other food items, and coatings or edible films (SDG 2). Finally, the review alludes to the safety concerns related to the utilization of edible flowers, thus offering an integrated dialogue on their advantages as well as possible applications. All of the aforementioned SDGs have been addressed in the following parts and sub-sections.

## An overview of flower polyphenols

Polyphenols are a broad group of secondary metabolites obtained from plants, biosynthetically formed from the shikimate pathway, and defined by the presence of aromatic moieties including hydroxyl groups (Sotto and Giacomo 2023). The principal polyphenolic groups include flavonoids, lignans, tannins, and stilbenes; in addition, additional phenolic compounds, such as phenolic acids (cinnamic acid, gallic acid, and caffeic acid), which are created by the same cascade as other chemicals, compose this class (Sotto and Giacomo 2023). The health benefits of certain polyphenols derived from edible flowers are listed in Table 1. Navarro et al. (2015) conducted high-performance liquid chromatography using diode array as well as mass detectors (HPLC–DAD/MS) analysis and revealed that amongst the characterized flavonoids in the flowers of *Tagetes erecta*, *Spilanthes oleracea*, and *Tropaeolum majus*, the primary ingredients were flavanol glycosides such as quercetin-rhamnosyl-rutinoside, quercetin-deoxyhexoside-di-hexoside, isorhamnetin-3-O-hexoside, and kaempferol-O-acetylhexoxide. The phytochemical profile of *Viola tricolor* flowers indicates that derivatives of flavonol-3-O-glycosyl are the primary elements of the flowers (Koike et al. 2015). Luteolin, hesperidin, acacetin, chrysoeriol, apigenin, and their derivatives represent the primary flavones identified in edible flowers. For instance, acacetin-7-O-(6-O-malonyl-O-glucoside, at a concentration of 1.41 mg/g of fresh weight (FW), has been detected in *Chrysanthemum morifolium*. Additionally, chrysoeriol-7-O-β-D-glucopyranoside, present at 0.2 mg/g dry weight (DW), and chrysoeriol at 0.24 mg/g DW have also been noted in *Lonicera japonica* (Janarny et al. 2021). Zheng et al. (2019) undertook a study encompassing 70 edible flower species in China, revealing that cyanidin-3-glucoside is the predominant anthocyanin present in the examined edible flowers. Furthermore, Zhao and colleagues (2015) documented that the occurrence of peonidin and pelargonidin derivatives in the flowers of *Paeonia suffruticosa* contributes to the reddish-purple and pinkish–blue hues of their petals. The purple–violet coloration observed in transgenic flowers of *C. morifolium* is primarily ascribed to anthocyanins such as delphinidin 3-O-(3″,6″-dimalonylglucoside) and delphinidin 3-O-(6″-malonylglucoside) (Iwashina 2015).

**Table 1:** Polyphenols isolated from various edible flowers along with their health benefits

Flower verity common/scientific name	Polyphenols	Biological activities	References
Snapdragon/*Antirrhinum majus* L.	**Flavonoids**: Pelargonidin-rhamnosyl-glucoside, anthocyanidins, quercetin-3-O-glucoside, kaempferol-3-O-rutinoside, cyanidin-rhamnosyl-glucoside, kaempferol-3-O-glucoside; **Phenolic acids**: NS; **Stilbenoids**: NS	Anticancer activities	Janarny et al. (2021), Seo et al. (2020), González et al. (2018)
Hybrid tuberous begonia/*Begonia x tuberhybrida*	**Flavonoids**: Catechin, epigallocatechin gallate, procyanidin B1, procyanidin B2, naringenin, quercitin3-Glucoside, kaempferol 3-glucoside, myricetin, Malvidin3,5-diglucoside; **Phenolic acids**: p-coumaric acid; **Stilbenoids**: cis-resveratrol, trans-resveratrol	Antioxidant activities	Janarny et al. (2021), Mlcek et al. (2021), de Morais et al. (2020)
Paper flower/*Bougainvillea glabra*	**Flavonoids**: Catechin, epicatechin, rutin; **Phenolic acids**: p-hydrobenzoic acid, gallic acid, sinapinic acid; **Stilbenoids**: NS	Antimicrobial activities	García et al. (2023), Janarny et al. (2021), Saleem et al. (2020)
Florist’s daisy/*Chrysanthemum morifolium* ramat	**Flavonoids**: Hesperetin, apigenin, chrysoeriol; **Phenolic acids**: Caffeoylquinic acid; **Stilbenoids**: NS	Antibacterial, antioxidant anti-inflammatory, neuroprotective, cardiovascular activities	Liang et al. (2025), Sharma et al. (2023), Pires et al. (2021)
Pansy/*Viola wittrockiana*	**Flavonoids**: Delphinidin-rhamnosyl- Glucoside, cyanidin − 3-(coumaroyl)-methylpentosyl-hexosyl − 5-hexoside, quercetin, kaempferol; **Phenolic acids**: Gallic acid, chlorogenic acid, caffeic acid, p-coumaric acid; **Stilbenoids**: NS	Antioxidant activities	Kozicka and Hallmann (2023), Janarny et al. (2021)
Mini rose/*Rosa chinensis* Jacq.	**Flavonoids**: Catechin, epicatechin, epicatechin gallate, epigallocatechin gallate, procyanidin B1, procyanidin B2, procyanidin A2, hesperidin, naringenin, quercitin 3-glucoside, rutin, kaempferol 3-glucoside, pelargonidin 3,5-diglucoside; **Phenolic acids**: Caftaric acid, chlorogenic acid, caffeic acid, syringic acid; **Stilbenoids**: cis-resveratrol, trans-resveratrol	Antioxidant, antibiosis activities	Zhou et al. (2023), de Morais et al. (2020)
Roselle/*Hibiscus sabdariffa*	**Flavonoids**: Quercetin − 3-O-sophoroside, quercetin 3-glucoside, myricetin3-sambubioside, cyanidin − 3-O-sambubioside, delphinidin − 3-sambubioside; **Phenolic acids**: Gallic acid; **Stilbenoids**: NS	Anti-inflammatory, antimicrobial activities	Almajid et al. (2023), Janarny et al. (2021)
Drumtstick/*Moringa oleifera* Lam	**Flavonoids**: Quercetin, kaempferol, naringenin, naringin, catechin, myricetin; **Phenolic acids**: O-coumaric acid; **Stilbenoids: R**esveratrol	Antioxidant, neuroprotective, antimicrobial activities	Gao et al. (2025), Kumar et al. 2025a), Prabakaran et al. (2018)
Pomegranate/*Punica granatum* Linn	**Flavonoids**: Apigenin − 7-O-glucoside, luteolin-O-diglucoside, cyanidin − 3-glucoside, pelargonidin − 3-O- glucoside; **Phenolic acids**: Gallic acid; **Stilbenoids: NS**	Anti-diabetic activities	Maphetu et al. (2022), Janarny et al. (2021)
Mountain ebony/*Bauhinia variegata*	**Flavonoids: E**picatechin, rutin, quercetin, luteolin; **Phenolic acids**: Gallic acid, protocatechuic acid, vanillic acid, caffeic acid, syringic acid, p-coumaric acid; **Stilbenoids**: NS	Antioxidant activities	Hegde et al. (2023)
Big marigold/*Tagetes erecta*	**Flavonoids**: Quercetin, luteolin; **Phenolic acids**: Gallic acid, protocatechuic acid, vanillic acid, caffeic acid, syringic acid, p-coumaric acid; **Stilbenoids: NS**	Antioxidant activities	Hegde et al. (2023)
Burans/*Rhododendron arboreum*	**Flavonoids**: Luteolin, diosmin, epigallocatechin 3-gallate, petunidin-3-glucoside; **Phenolic acids**: Ellagic acid, caffeic acid 3-glucoside; **Stilbenoids**: NS	Antioxidant, anticancer, anti-mutagenic activities	Sendri et al. (2024), Bhatt et al. (2022), Gautam et al. (2020)

NS: Not specified

Gallic acid, p-hydrobenzoic acid, syringic acid, protocatechuic acid, chlorogenic acid, and their derivatives were the main phenolic acids discovered in edible flowers (Janarny et al. 2021). The phenolic acids were documented in flowers of *Cassia siamea*, *Tagetes erecta*, *Cosmos sulphureus*, *Taraxacum officinale*, *Bellis perennis*, *Tragopogon pratensis*, *Trifolium repens*, *Dahlia mignon*, and *Rumex acetosa* (Moliner et al. 2018; Kaisoon et al. 2011). Caffeyolquinic acid was reported in different forms, viz., 5-caffeyolquinic acid, 4-caffeyolquinic acid, and 3-caffeyolquinic acid in the *Prunus mume* and *Hibiscus sabdariffa* flowers and 3-caffeyolquinic acid and 1-caffeyolquinic acid in flowers of *Chrysanthemum morifolium* (Ramirez et al. 2011; Shi et al. 2009).

### Flowers derived carbon dots (CDs) (SDG 6)

Carbon dots (CDs) are < 10 nm in size and have a quasi-spherical structure (Batool et al. 2022). The carbon atoms in CDs exist in sp^2^ hybridization along with some sp^3^centers (Batool et al. 2022). CDs have received significant interest because of their outstanding ability to remain stable under light, low toxicity levels, and compatibility with living beings, water dissolving capacity, high sensitivity, good emission ability, capability in selectively detecting specific substances, huge difference between wavelengths of absorbed and emitted light, and ability of adjusting light emission wavelengths (Meng et al. 2019). Biomass with a significant concentration of heteroatoms is regarded best feedstock for the synthesis of carbon dots, as contrasted to CDs formed from synthetic carbon sources, which necessitates the incorporation of several external heteroatoms (Meng et al. 2019). The production of CDs from a range of natural resources and their possible uses in fluorescence sensing of a broad range of pesticides have recently been effectively proven in a number of studies (Vadia et al. 2023; Kailasa and Koduru 2022; Yusuf et al. 2022). Because they are environmentally friendly and sustainable, green source-based CDs are crucial for implementing green chemistry (Vadia et al. 2024).

**Fig. 1:**
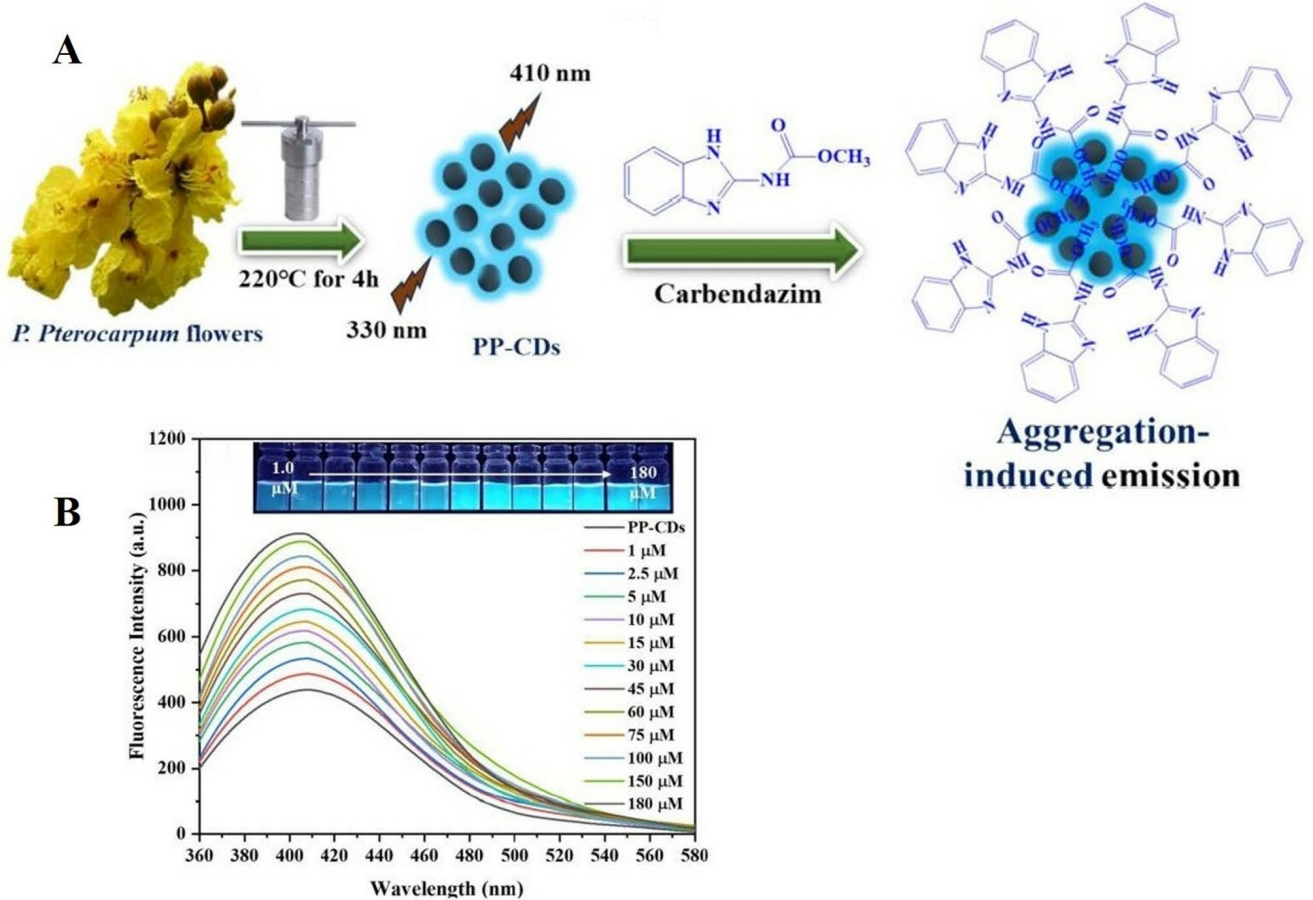
**A** Synthesis of carbon dots using flowers of *Peltophorum pterocarpum* as biomass for the detection of carbendazim (CBZ) **B** Emission spectra of flower derived carbon dots after adding various concentrations of carbendazim (Vadia et al. 2024; License number 6021451448154)

Vadia and co-workers (2024) have synthesized CDs from *Peltophorum pterocarpum* flowers by the hydrothermal method, acting as precursor material. The synthesized CDs served as a potential fluorescent probe for the detection of carbendazim (CBZ) through an emission mechanism induced by aggregation, displaying a linear response towards CBZ concentrations in the range of 1–30 µM and with a limit of detection of 5.41 nM (Fig. 1). Another set of CDs has been successfully synthesized for the very first time from the flowers of *Plumeria alba* serving as raw material and prepared by a green and low-cost hydrothermal method, and displayed good water solubility (He et al. 2023). The researchers have used sodium hydroxide and ammonia to regulate the luminescence of prepared CDs. Their findings showed that the blue fluorescent carbon dots synthesized using ammonia as solvent were utilized for specific identification and detection of Cu^2+^ in water, while the green fluorescent CDs prepared from sodium hydroxide as solvent were employed for detection of pH. Huang and teammates (2019) have reported that the carbon dots synthesized using flowers of cherry blossom showed bright blue fluorescence when observed under UV irradiation, thus making them appropriate for fluorescent ink. In another study, fluorescent CDs were prepared by using *Borassus flabellifer* (male tree) flowers through thermal pyrolysis, serving as an efficient probe to detect Fe^3+^ ions in a range of 0–30 nM and having a 10 nM limit of detection (Murugan and Sundramoorthy 2018). The biggest issue encountered while producing CDs from biomass is the unpredictability of output, which can swing between low and high based on variances in their composition (Naziba et al. 2024).

## Flowers derived metallic nanoparticles (MNPs) (SDG 3, and 6)

The term ‘nanoparticles’ is derived from the Greek word ‘nano’, which means small or dwarf. It is also used as a prefix to indicate a size of one billionth of one meter, or 10–9, which translates to 1 nm (Jamkhande et al. 2019). Noble metals, such as platinum, silver, and gold, have good impacts on human health and are thus used to synthesize nanoparticles, which have become known as metallic nanoparticles (Jamkhande et al. 2019). The composition and quality of natural components employed in the production of nanoparticles, also known as biogenic synthesis or phytosynthesis, are critical to achieving efficient and ecologically beneficial results. The procedure for producing metal oxide nanoparticles involves an interaction between an appropriate biological extract and a metal salt solution. Certain chemicals found in biological extracts operate as capping agents, helping to stabilize nanoparticles and facilitate nanofabrication operations (Tolisano and Buono 2023). Furthermore, biological extracts can convert metallic precursors to a zero-valence state. Precursor reduction stabilizes the precursors and ensures nanoparticle nucleation (Rabalao et al. 2022). The produced nanoparticles have the potential to enhance a variety of areas, including health, food, agriculture, and the environment, lending support to the circular economy idea. Table 2 shows a collection of many studies and their conclusions relevant to the research activity. The research mostly covered looked at the method of collecting MNPs, reviewing their health impact, and evaluating the environmental consequences of MNPs synthesized from diverse flowers.

**Table 2:** MNPs derived from different flowers along with their applications

Flower scientific name	Common name	Type of MNPs synthesized	Size (nm)	Shape	Applications	References
*Tecoma stans*	Yellow elder	Copper oxide (CuONPs)	30.47	Spherical	Antibacterial activity against *Pseudomonas aeruginosa* and *Bacillus subtilis*; anticancer activity towards lung cancer cells (A549); degradation of dye, methylene blue (MB) up to 88%	Vasantharaj et al. (2024)
*Bougainvillea glabra*	Paper flower	Copper oxide	36 –54	Cylindrical	Strong photocatalytic effectiveness displayed in liquid media wherein oxidation of bromothymol blue, 4-nitrophenol and Congo red takes place in an acidic acetic anhydride solution	Natrayan et al. (2023)
*Aglaia elaeagnoidea*	Priyangu	Copper oxide	20 –45	Spherical	High catalytic activity to reduce Congo red, 4-nitrophenol and methylene blue at room temperature and with sodium borohydride (NaBH_4_) present in the solution; CuONPs catalyst can easily be recovered by centrifugation, with subsequent reuse for 6 cycles and conversion efficiency greater than 90%	Manjari et al. (2017)
*Calendula officinalis*	Pot marigold	Gold (AuNPs)	20−25	Semi-spherical	Therapeutic potential against diabetes-induced cardiac dysfunction observed in rat	Hao et al. (2022)
*Crocus sativus*	Saffron crocus	Gold	25	Spherical, and oval	Antibacterial activity towards *Escherichia coli*; significant activity inhibition of enzyme urease	Alhumaydhi et al. (2021)
*Mangifera indica*	Mango	Gold	10 –60	Spherical	AuNPs exhibited brilliant nanocatalysis while reducing 4-nitrophenol to 4-aminophenol in the presence of NaBH_4_ in the aqueous phase	Nayan et al. (2018)
*Plumeria alba*	White frangipani	Gold	15.6 –28	Spherical	Antibacterial activity towards *Escherichia coli*; 4-nitrophenol dye was rapidly degraded into 4-aminophenol within five minutes	Mata et al. (2016)
*Carthamus tinctorius* L.	Safflower	Gold	40 –200	Triangle, and spherical	Antibacterial activity towards *Escherichia coli*	Nagaraj et al. (2012)
*Gnidia glauca*	Balsam	Gold	10	Spherical	AuNPs exhibited significant catalytic properties in the reduction of 4-nitrophenol into 4-aminophenol in the presence of NaBH_4_ in an aqueous phase	Ghosh et al. (2012)
*Nymphaea tetragona*	Pygmy waterlily	Platinum (PtNPs)	2 –4	NS	PtNPs can inhibit biosynthesis of UVB-induced melanin and tyrosinase activity significantly in A375 human melanogenic cells	Zhang et al. (2023)
*Cassia fistula* L.	Golden shower	Silver (AgNPs)	50 –100	Spherical	AgNPs showed potential in quenching unstable cation free radicals on ABTs (67.79%), nitric oxide (64.15%), and DPPH (60.34%), depending upon concentration co-relatedness; The NPs triggered pathological structural cellular damage in *Staphylococcus aureus* and *Escherichia coli*; AgNPs exhibited photocatalytic efficiency in degradation of crystal violet (65.14%) and methylene blue (83.82%) dyes	Omran (2024)
*Bougainvillea glabra*	Paper flower	Silver	10 –50	Spherical, and oval	Anticancer activity against lung cancer cells (A549); Antibacterial activities against *Staphylococcus aureus* and *Escherichia coli*; Antioxidant activity determined using DPPH	Oves et al. (2023)
*Jasminum nudiflorum*	Winter jasmine	Silver	13	Cubic	Antifungal activity exhibited against *Alternaria longipes*; Determined antioxidant activity by using DPPH	Yang et al. (2023)
*Aerva lanata*	Mountain knotgrass	Silver	5 –15	Spherical	The biosynthesized AgNPs displayed antibacterial activity towards Gram-negative and Gram-positive bacteria; DPPH assay revealed antioxidant activity of AgNPs at 100 mg/mL concentration; Photocatalytic activity of AgNPs was analysed and confirmed by Congo red and cotton blue dye degradation/decolorization activity by reducing 4-nitrophenol to 4-aminophenol	Palithya et al. (2022)
*Couroupita guianensis* Aubl.	Cannonball tree	Silver	34	Spherical	The biosynthesized AgNPs showed considerable free radical scavenging potential using DPPH antioxidant assay; Silver nanoparticles also showed strong antibacterial activity against numerous pathogenic bacterial species	Singh et al. (2021)
*Rosa damascena*	Damask rose	Silver	18 –40	Spherical	Good antibacterial activity towards *Staphylococcus aureus*	Peron et al. (2021)
*Plumeria pudica* Jacq.	Gilded spoon	Silver	27.1	Spherical	Displayed high antibacterial activity towards *Escherichia coli*; DPPH, ABTS, and superoxide assays used free radical scavenging, and inhibitory concentration (IC_50_) values of 30.4 µg/mL, 32.5 µg/mL, 30 µg/mL and 18 µg/mL were observed; AgNPs showed significant a dose-dependent cytotoxic potential against human liver cancer cells (HepG2) with 62.07 µg/mL IC_50_ value and induced a time-dependent ROS production followed by apoptosis; a significant larvicidal potential of AgNPs was observed against *Culex quinquefasciatus* having 0.79 µg/mL IC_50_ value	Suriyakala et al. (2021)
*Zephyranthes rosea*	Rain lily	Silver oxide (Ag_2_ONPs)	10 –30	Spherical	Ag_2_ONPs derived from *Zephyranthes rosea* were observed to be potential candidate for antibacterial activity against various strains of *Streptococcus mutants*, *Staphylococcus aureus*, and *Escherichia coli*; Antioxidant activity of theAg_2_ONPs was observed at 100 µg/mL and was reported as 73.82% using ascorbic acid as standard drug. Anti-inflammatory activity of nanoparticles was recorded at 500 µg/mL and was reported as 97.19% using BSA denaturation technique; Anti-diabetic activity of Ag_2_NPs was tested using α-amylase assay and inhibition percentage at 500 µg/mL was reported as 75.7%	Maheshwaran et al. (2020)
*Mangifera indica*	Mango	Silver	10 –20	Spherical	The AgNPs displayed widespread lethal effect on the Gram-negative (*Pantoea agglomerans*, *Rahnella* sp., and *Klebsiella* sp.) and Gram-positive (*Staphylococcus* sp.) bacteria	Ameen et al. (2019)
*Brassica oleracea italica*	Broccoli	Zinc (ZnNPs)	100 –500	Spherical	Observed good antifungal activity against *Paeciolmyces fulvus*	Khan et al. (2021)
*Syzygium aromaticum*	Clove	Zinc oxide (ZnONPs)	30 –40	Triangular and hexagonal	Synthesized ZnONPs reduced production and growth of zearalenone and deoxynivalenol in *Fusarium graminearum* in a broth culture	Lakshmeesha et al. (2019)

NS: Not specified, DPPH: 2,2-diphenyl-1-picrylhydrazyl, ABTS: 2,2’-azino-bis(3-ethylbenzothiazoline-6-sulfonic acid), ROS: Reactive oxygen species, UVB: Ultraviolet B, BSA: Bovine serum albumin

Recently, Velmurugan et al. (2024) have investigated cupric oxide nanoparticles (CuONPs) prepared using the flower extract of *Jasminum sambac* and found it to be efficient as a catalyst in breaking down the dye, methylene blue (MB), on exposure to solar and UV radiation. Furthermore, the researchers demonstrated a pH-dependent breakdown of the dye. The findings of the study suggest that the best concentration of dye to start complete breakdown of components is estimated to be 2.5 parts per million (ppm), thus resulting in a remarkable disintegration rate of 79%. Flower extract of *Couroupita guianensis* based CuNPs turned out to be effective in the inactivation of *Escherichia coli*, thereby achieving 2% inactivation at 0 min, 52% at 30 min, and 99% at 60 min, and with *Listeria monocytogenes*, achieved 1% inactivation at 0 min, 48% at 30 min, and 98% at 60 min, under visible light. Additionally, the CuNPs displayed significant anticancer potential against A549 cells or human non-small cell lung cancer (NSCLC) cells, and achieved cell viability reductions starting from 10% at 25 µg/mL concentration, 30% at 50 µg/mL, 50% at 100 µg/mL, 70% at 150 µg/mL, 83% at 200 µg/mL, to 91% at 250 µg/mL (Saritha et al. 2024). Saha and co-workers (2025) have reported that the flower extract of *Madhuca indica*-derived gold nanoparticles (AuNPs) showed strong anticancer activity against head and neck squamous cell carcinoma (HNSCC) cell lines. The anticancer potential was evident from a decrease in CD44+/CD24- subpopulation and tumor sphere formation efficiency alongside dose-dependent downregulation of epithelial to mesenchymal transition (EMT) markers and pertinent cancer stem cells (CSC) markers expression in both ex vivo and in vivo HNSCC model. Another study on AuNPs decorated with *Nyctanthes arbor-tristis* flower (NAF) displayed significant antibacterial efficacy towards *Pseudomonas aeruginosa* (Sobi et al. 2024).

Flowers of *Clitoria ternatea* L. were used to green synthesize silver nanoparticles (CT-AgNPs), focusing on their antimicrobial and anticancer properties (Singh et al. 2025). Their findings revealed a dose-dependent cytotoxic action on the human alveolar basal epithelial cells (A549), having an LC_50_ value of 4.88 µg/mL, whereas the CT-AgNPs showed no significant toxicity against human embryonic pulmonary epithelial cells (L-132) up to a dose of 30 µg/mL. In addition, the antibacterial activity of CT-AgNPs was estimated using survival assays of *Caenorhabditis elegans*, where they significantly decreased the paralysis rates and enhanced nematode survival on exposure to pathogenic bacteria such as *Staphylococcus aureus* and *Pseudomonas aeruginosa* at 20 µg/mL and 40 µg/mL concentrations. Barabadi et al. (2024) have documented the synthesis of AgNPs using *Punica granatum* flower aqueous extract and found that these exhibited antibacterial potential at molecular and phenotypic levels against *Acinetobacter baumannii* and *Staphylococcus aureus* with 16 µg/mL and 32 µg/mL minimum inhibitory concentration (MIC) values, respectively. Zinc oxide nanoparticles (ZnONPs) derived from the flower extracts of *Butea monosperma* displayed strong antibacterial action against *Bacillus subtilis*, *Klebsiella pneumoniae*, and *Escherichia coli* (Alam et al. 2025).

Dikshit et al. (2021) identified specific challenges encountered during green synthesis of MNPs, including extensive optimization research on process parameters (pH, rotational speed, temperature, etc.) and reactants (particularly plant-based materials) required for MNP shape and size regulation. To improve MNP yield and stability while lowering reaction time, a variety of reaction parameters must be optimized.

## Flower-derived biochar (SDG 6, and 9)

Biochar constitutes a carbon-rich material produced from the organic feedstock by subjecting it to thermal combustion under limited oxygen supply (Wang and Wang 2019). Another study by Mishra and Mohanty (2023) documents that biochar primarily originates from the pyrolysis of biomass. Biochar production is economical (lower energy loss and higher product yields), environmentally benign (biomass is carbon neutral due to carbon dioxide emissions from biomass, which are offset by carbon assimilation during biomass photosynthesis; it is also shown to have fewer negative effects on the atmosphere due to lower levels of sulfur (S) and nitrogen (N), which result in lower emissions of sulfur oxides (SOx) and nitrogen oxides (NOx) compared to fossil fuels), and effective for reusing waste materials (Cha et al. 2016). This strategy can expedite the required shift towards a carbon-neutral economy (Sani et al. 2023). Therefore, biochar generated from waste can provide a substitute methodology for the management of garbage and recovery of resources, thereby facilitating a reduction in climate change and safeguarding the environment.

Subsequently, greater human involvement has increased quantities of metals that induce toxicity in water, posing a serious environmental danger to aquatic ecosystems. Though the scarcity of water for agriculture has resulted in a rethinking of innovative technologies for wastewater treatment (Kumar et al. 2025b). Numerous studies have consistently demonstrated that the incorporation of biochar into wastewater may enhance its quality while also efficiently removing heavy metals and drug residues from contaminated drinking water (Mishra et al. 2023; Ai et al. 2020; Cheng et al. 2020; Shakya et al. 2019).

Samal and researchers (2025) have examined the facile fabrication of a novel composite (magnetic) by embedding flower biochar of *Neolamarckia cadamba* with polypyrrole (Ppy@MNCFB) and iron (II, III) oxide (Fe_3_O_4_) for chromium (Cr(VI) adsorption in simulated as well as spiked real wastewater. The results displayed that the material had minimal Cr(VI) adsorption inhibition when present with spiked wastewater and coexistent ions, thus supporting its field applicability. After recycling, the exhausted material was pyrolyzed to overcome the long-term problem of solid disposal while establishing a secondary adsorbent having 51% efficiency. Cement replacement with palm flower biochar (PFC) at 1% cement weight aids in enhancing various properties of porous concrete without compromising the mix’s compressive strength (Abhinaya et al. 2023). With the PFC content, improvement in the sample’s water purification ability was also witnessed. Gautam et al. (2025) have developed bougainvillea flowers (BG)-derived biochar. Results have shown that the biochar, which underwent chemical activation treatment, displayed significantly increased surface functional groups and porosity. The biochar (BG-H_3_PO_4_) treated with phosphoric acid (H_3_PO_4_) demonstrates superior performance in comparison to both untreated BG-biochar and potassium hydroxide (KOH) treated one (BG-KOH) using zinc-ion hybrid super-capacitors (ZIHSCs). Biochar produced after pyrolysis has been shown to lack enough porosity and surface functional groups by Mishra and Mohanty (2023). Therefore, before employing biochar as a robust and effective functional solid for a variety of real-world applications, it is crucial to expose it to surface functionalization. The porosity and surface area of biochar may be increased using a variety of physicochemical techniques, which will increase its potential uses in energy storage devices, nanotubes, bio-composites, environmental protection, catalysis, and supercapacitors (Kumar et al. 2024c).

## Flower-derived bio-adsorbent (SDG 6)

The elimination of contaminants, such as organic, inorganic, and biological pollutants, can be aided by adsorption techniques (Saravanan et al. 2023). According to Aguilar et al. (2022), biosorption is the process by which a pollutant or adsorbate is held to the surface of an adsorbent (biomaterial), forming a molecular or atomic film as a result of lingering interactions such as covalent bonds or Van Der Waals forces. Because of its many unique benefits, activated carbon (AC) has attracted a lot of interest in the adsorption process. It is very effective for a variety of industrial and environmental applications due to its high surface area, which is the consequence of its porous structure and offers a large number of binding sites for adsorbate molecules (Sudarsan et al. 2025). The qualities required for a great adsorbent include stability, low cost, high sorptive capacity, and non-polluting nature. Current studies have focused on affordable adsorbents and related processes to eliminate pollutants, increasing the removal capability to 99.99% (Saravanan et al. 2023). Overall, employing readily available and reasonably priced biological materials, such as biomass, microbial cultures, or agricultural waste, can significantly reduce the cost of the adsorption process (Saravanan et al. 2023). There are several advantages of using biomass waste to create bio-adsorbents, including high efficiency, low cost, ease of synthesis, sustainability, renewability, high surface area, functional groups, low toxicity, pH and temperature tolerance, or surface modification (Saravanan et al. 2023; Wong et al. 2018). To meet cleaner production targets and treat wastewater, the circular economy idea has made it easier and more efficient to use waste resources and reprocess them (Hossain et al. 2020). Flavonoids, tannins, lignin, and other bioactive substances are among the phytochemicals that are particularly abundant in flowers, forming a large number of active sites for effective adsorption (Sudarsan et al. 2025). As will be covered in the next paragraph, researchers from all over the world have been actively investigating the application of bio-adsorbents made from various flowers for wastewater treatment.

**Fig. 2:**
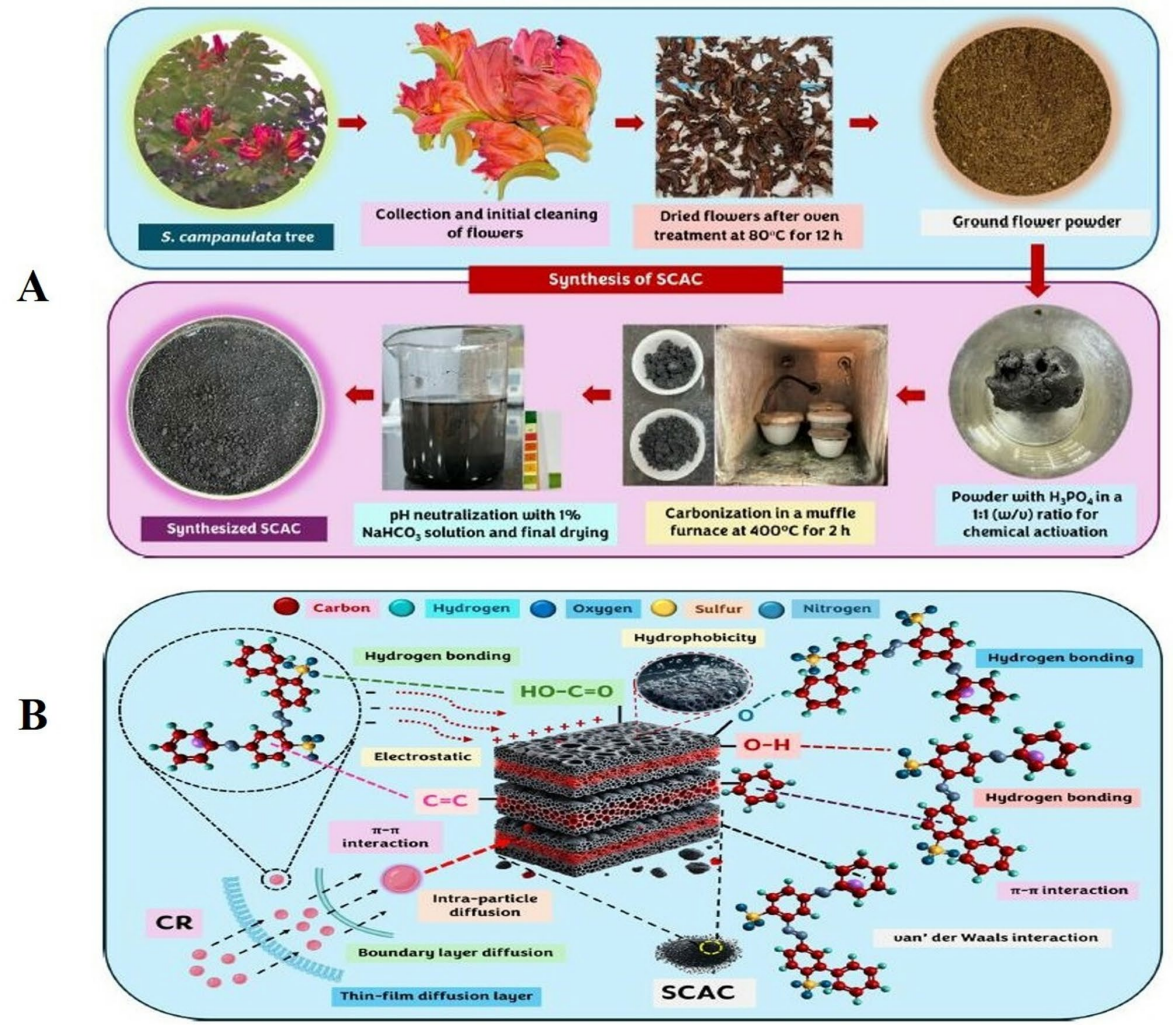
**A** Graphical presentation of the synthesis bio-adsorbent using *Spathodea campanulate* flowers **B** Key interactions between flower derived bio-adsorbent and Congo red (CR) dye during adsorption (Sudarsan et al. 2025; under a Creative Commons Attribution 4.0 International License)

Sudarsan and researchers (2025) have investigated the synthesis, characterization, and possible application of *Spathodea campanulata* flowers-derived activated carbon (SCAC) to remove dye, Congo red (CR), from aqueous streams. The results suggested a monolayer adsorption mechanism having a 59.27 mg/g maximum adsorption capacity (Fig. 2). Desorption studies displayed methanol to be the most effective desorbent, with SCAC retaining significant adsorption capacity across 6 cycles, thus highlighting its reusability. Another study by Vemula et al. (2023) has reported that bio-adsorbent prepared from *Peltophorum pterocarpum* flowers (PPF) shows maximal removal adsorption efficiency of 71.9434 mg/g for methylene blue (MB), and the pseudo-second-order model was appropriate for adsorption kinetics. In a batch experiment, Srinivasulu (2023) demonstrated that *Senna auriculata* L. flower petal biomass, as a bio-adsorbent, was able to remove 80% fluoride at a sorbent dose of 0.25 g/100 mL, pH 6, 90 min of agitation time, and an initial fluoride ion concentration of 5 mg/L. When applied to actual field water samples that were tainted with fluoride, the manufactured biosorbent proved to be successful. Another study was conducted using batch mode adsorption to investigate dried bael flowers of *Aegle marmelos* for its adsorption capacity to remove Cu(II) ions from the aqueous solutions by changing agitation time, dose of adsorbent, initial metal concentration, initial pH and temperature of the copper ion solution (Thanthri et al. 2021). Results revealed that the Langmuir isotherm model showed a good agreement with experimental data, having a correlation coefficient (R^2^) of 0.97, and the maximal adsorption capacity achieved was 23.14 mg/g recorded at 30 °C. The sorption thermodynamic study results suggested that the process of adsorption is spontaneous and exothermic.

## Essential oils from various flowers (SDG 8)

Essential oils (EOs), which are secondary metabolites isolated from flowers, consist of aromatic and volatile constituents extracted from various edible flowers utilizing diverse extraction methods such as solvent extraction, microwave-assisted extraction, supercritical fluid extraction, and steam distillation (Chen et al. 2021). These oils have been extensively utilized in the fields of medicine and cosmetics due to their fresh, sweet-smelling, and fragrant characteristics, in addition to their pharmacological properties (Ksouda et al. 2019; Hussain et al. 2016). Owing to the abundance of active components, EOs derived from flowers exhibit antioxidant, anti-pest, and antimicrobial activities, which confer a significant fresh-keeping effect on food products (Chen et al. 2021) (Table 3).

**Table 3:** Essential oil extracted from various flowers

Flower scientific name	Common name	Extraction method	Yield	Biological activities	References
*Psidium cattleianum* Sabine	Strawberry guava	Supercritical fluid extraction (SFE)	1.4%	Anti-inflammatory activity by inhibition of COX-2 enzyme	Elsayed et al. (2023)
*Paeonia delavayi*	Tree peony	Supercritical carbon dioxide	0.78% (dark-purple petals), 0.64% for red petals), and 0.58% for yellow petals)	Observed antioxidant activities	Yu et al. (2022)
*Calendula officinalis* L.	Pot marigold	Hydrodistillation using a Clevenger-type apparatus	NS	Essential oils were observed to possess antioxidant potential using reducing and free radical scavenging mechanisms; furthermore, the extracted essential oil inhibited all the enzymes tested, namely amylase, acetylcholinesterase, butyrylcholinesterase, tyrosinase, and glucosidase	Ak et al. (2021)
*Laurus nobilis* L.	Bay tree	Hydrodistillation using a Clevenger-type apparatus	1.06%	Essential oil isolated from flowers of *L. nobilis* exhibited significant antifungal potential against *Aspergillus niger*, A. *clavatus*,* Chaetomium globosum*, *Myrothecium verrucaria*, *Cladosporium cladosporioides*, *Trichoderma viride* and *Penicillium citrinum*; high total antioxidant capacity (TAC) was indicated by its capability of scavenging DPPH free radical	Mssillou et al. (2020)
*Calendula officinalis* L.	Pot marigold	Hydrodistillation using a Clevenger-type apparatus	0.1%	Showed strong antibacterial activity against Gram-negative and Gram-positive bacteria	Sahingil (2019)
*Lavandula angustifolia*	Lavender	Hydrodistillation	1.55 g/100 g (fresh flowers), and 2.87 g/100 g (dried flowers)	Showed high antibacterial activity against bacteria (*Staphylococcus aureus*, *Bacillus subtilis*, *Pseudomonas aeruginosa*,* Escherichai coli*), filamentous fungi and yeast (*Aspergillus niger*,* Candida sp.*,* Penicillium expansum*)	Smigielski et al. (2018)
*Matricaria chamomilla* L.	Chamomile	Hydrodistillation	0.5%	Displayed antioxidant activity	Stanojevic et al. (2016)
*Callistemon citrinus*	Scarlet bottlebrush	Hydrodistillation using a Clevenger-type apparatus	0.12%	Cytotoxicity on lung cancer cells (A549)	Kumar et al. (2015)

NS: Not specified, COX-2: Cyclooxygenase-2, DPPH: 2,2-diphenyl-1-picrylhydrazyl

Flowers EOs consist of nearly 20–60 components, mainly categorized as terpenes, terpenoids, along with aromatic constituents based on their chemical structure. Amongst these components, numerous major constituents are found at relatively higher percentages while others are present in small amounts (Burt 2004). The components of floral essential oils mostly depend on the species (Ribeiro et al. 2017). Rassem and co-workers (2022) have used the conventional microwave-assisted hydrodistillation (MAHD) and hydrodistillation (HD) to extract essential oils from *Hibiscus rosa-sinensis* or hibiscus flower, abundant in oxygenated monoterpene, sesquiterpene hydrocarbons, and sesquiterpene. *Prunus avium* L., or sweet cherry flower essential oil, consists of ethanol, lilac alcohol, linalool, acetaldehyde, benzaldehyde, dimethyl sulphide, and (*E*)-2-hexenal as the main volatiles, which primarily contribute to the characteristic sweet cherry flower aroma (Zhang et al. 2021). Indeed, Park and teammates (2019) have reported that the major components of downy lavender flower EO were p-cymenene (11.66%), 1-methyl-6-isopropylidene (14.94%), 4-terpinyl acetate (16.99%), and 1,3-dimethyl-1,5-cyclooctadiene (19.7%), whereas thymol (8.45%), germacrene (11.82%), and linalool (29.15%) were the major components of *Cananga odorata* flower oil (Kalagatur et al. 2018). Camphor (11.39%), L(-)-borneol (14.90%), and β-eudesmene (18.17%) made up the majority of the essential oil extracted from *Chrysanthemum flos* flowers (Lin et al. 2019). The main flower sources for various aromatic compounds like eugenol, cinnamaldehyde, methyl eugenols, and benzyl benzoate are clove, iris, ishpingo, myrtle, and the *Rhaponticum* genus. Terpenes like thymol, 1,8-cineole, carvacrol, linalool, p-cymene, camphor, and α-pinene are frequently found in flower essential oils extracted from flowers such as saffron crocus, lavender, chrysanthemum, thyme, ylang-ylang, and jasmine (Chen et al. 2021).

### Enrichment applications of edible flowers in food (SDG 2)

**Fig. 3:**
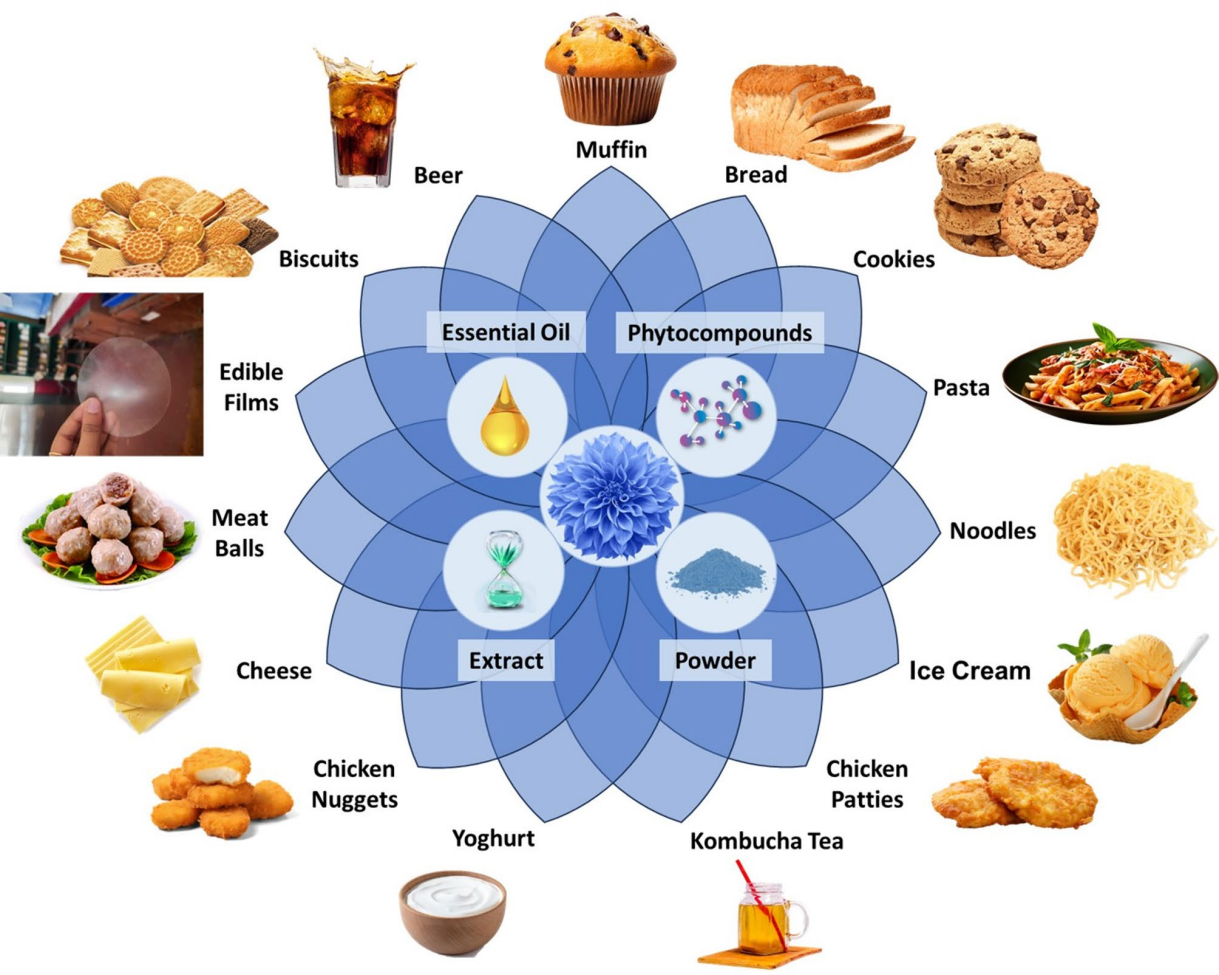
Formation of different types of functional foods enriched with various forms of edible flowers

Technological advancements have facilitated the integration of novel nutrient sources and natural additives, consequently resulting in enhanced functionality as well as the utilization of various by-products (Capozzi 2022). The concept of the circular economy has been demonstrated to be effective in the domain of innovative nutritional resources, exhibiting comparable or superior quality to traditional alternatives. Circular bioeconomy (CBE) is utilized due to its capability to harmonize the necessity for nutritious food with environmentally sustainable production methods (Capozzi 2022). A balanced diet should encompass a comprehensive array of micronutrients and macronutrients essential for meeting the nutritional requirements of human beings. Food enrichment is suggested as an intervention strategy in regions with a significant prevalence of micronutrient deficiencies, thus providing a framework for addressing the issue (Kumar et al. 2024c). Edible flowers abundantly contain phytochemicals and nutrients, offering several health benefits. When these flowers are used as food components, they provide a significant contribution to the food industry by utilizing easily available natural resources for the production of functional food items (Chetia et al. 2025). Edible flowers not only have a pleasing look and scent, but they also have good sensory and textural features when combined with other meal items. A burgeoning trend has been seen for the use of edible flowers in the production of culinary items, despite a lack of study behind it (Chetia et al. 2025). Figure 3 depicts a thorough overview of the improvements achieved in the creation of various food products containing edible flowers as extract, essential oils, powder, and phytocompounds.

### Cereal-based food

Since they include significant amounts of biomolecules like proteins and carbohydrates, meals made from various cereals, such as muffins, bread, biscuits, cookies, pasta, and noodles (Fig. 3), are an essential part of our daily dietary needs. But they are deficient in vitamins, minerals, fiber, phytochemicals, and other vital micronutrients. The increasing demand from consumers for nutrient-dense foods has led to the addition of beneficial elements to basic nutrition and health (Kumar et al. 2024c). Edible flowers are utilized as a functional component, typically in the form of powder, to provide dietary fiber in cereal-based food items (Table 4). *Opuntia ficus-indica* L., or prickly pear flower powder-derived biscuits, had the maximum anti-radical activity (81.04%). The sensory analysis disclosed biscuits formulated with 80% flowers had been preferred by the experts (Brahmi et al. 2024). In another study, marigold powder acted as the main source of lutein and was utilized for the production of lutein-fortified breads used for ocular health (Kwon et al. 2023). The results showed that the employment of marigold powder caused changes in extensional features and protein secondary structure of doughs, subsequently contributing to softer texture and increased loaf volume. The addition of saffron flower by-products, especially at a 10% concentration, raised the nutritional fiber content of spelt and typical wheat breads by 25% to 30%; increased the mineral content (Fe at 15–18 mg/100 g, Mg at 40–50 mg/100 g, Ca at 90–95 mg/100 g, and K at 270–290 mg/100 g); changed the textural characteristics; and significantly increased the phenolic content and antioxidant capacity (at 10 and 5%), which stayed constant during the gastrointestinal and in vitro oral digestion processes. The sensory analysis showed that the inclusion of saffron flowers to bread modified its organoleptic properties (Cerdá and Frutos 2023). Šťastná et al. (2021) have claimed that cookies containing kamut, dried mango, matcha tea, and jasmine were extremely loaded with flavonoids, specifically epigallocatechin and epicatechin. The prepared cookies could be recommended as an unconventional cookie to the bakery industry, owing to the health benefits they offer to consumers. According to Borczak and colleagues (2024), the muffins’ color, flavor, and perfume were all greatly affected when *Trifolium repens* L., or white clover flower flour, was substituted for wheat flour. Additionally, when more flour was added to the muffin dough, the amount of lipids, proteins, dietary fiber, resistant starch (RS), slowly digested starch (SDS), total polyphenols, total ash, and antioxidant activity all improved noticeably. The glycemic index value, in vitro melanoma cancer cell survival, and the amount of rapidly accessible glucose (RAG), quickly digestible starch (RDS), and free glucose (FG) all sharply declined on the same day. Likewise, muffins made with Roselle calyx extract have significant levels of antioxidant, phenolic, anthocyanin, and Cyn-3-glu (126.63 ± 1.96 mg/100 g), 12.53 ± 0.13%, 12.10 ± 0.89 mg/100 g, and 12.91 ± 0.69 mg gallic acid equivalents (GAE)/100 g, respectively (Marak et al. 2022).

**Table 4:** Milk and cereal-based food products developed using different edible flowers forms

Product developed	Form of flower used	Quantity added	Product quality	References
Biscuit	Drumtstick (*Moringa oleifera* Lam) powder	4, 3.25, 2.5, 1.75, and 1 part	The results displayed a calcium content of 115.73 mg/100 g that was greater in biscuits having 4 portions of flower powder; sensory analysis showed that biscuits having 1.75 portions of flower were deemed to be the best in terms of overall attributes assessed by panelists	Yadav et al. (2022)
	Date palm (*Phoenix dactylifera* L.) powder	0, 3, 6, and 9%	The findings revealed a significant increment (*p* < .05) in dietary protein (7.64–8.30%), ash content (1.30–1.68%), and fiber (1.22–84.81%) coupled with an increased darkness (77.30–58.52) as compared to control; sensory evaluation displayed that incorporation of powder up to 9% was positively correlated to different sensory attributes, with higher appreciation for the biscuits fortified at 6%	Karra et al. (2020)
Bread	Walnut (*Juglans regia* L.) powder	0.5, 1, 1.5, 2, and 2.5%	The use of additive at 2.5% caused significant value changes for most of the parameters examined for bread crumb texture; walnut male flower addition to the flour showed a considerable effect on the total polyphenol content and antioxidant potential of the tested breads and doughs	Pycia et al. (2022)
	Butterfly pea (*Clitoria ternatea* L.) extract	0.5, 1, and 2%	The flower extract addition significantly decreased the starch digestion rate for the wheat bread; the predicted glycemic index (pGI) for the bread containing 0.5% extract was significantly lesser than the control bread	Chusak et al. (2018)
Pasta	Black locust (*Robinia pseudoacacia* L.) powder	2.5, 5, 7.5, and 10%	Black lotus flower (BLF) incorporation increased the antioxidant activity and phenolic content; changes in the proximate composition and texture of the pasta having 2.5% BLF brought no changes in the consumer attractiveness for the obtained pasta	Kowalczewski et al. (2019)
Noodles	Butterfly pea (*Clitoria ternatea* L.) extract	10, 20, and 30%	Both cooked and dried noodles containing flower extracts at 20–30% showed a significantly high color preference in comparison to the control; blue noodles possessing high antioxidant activities, phytochemicals, and desirable sensory attributes can be prepared by the addition of flower extracts at 20–30%	Shiau et al. (2023)
Yogurt	Was mellow (*Malvaviscus arboreus*) extract	1, and 2%	Including extracts significantly increased the antioxidant activity of yogurt to a value of 10.17 µmol TEAC/g, along with strengthening its capability in inhibiting lipid oxidation on storage; sensory evaluations displayed positive results for formulations at 1 and 2%	Pontes et al. (2024)
	(*Rhododendron myrtifolium*) powder	1, and 2%	The results revealed that the color and texture of the prepared yogurt were greatly influenced by the inclusion of the powder; incorporation of this functional constituent also resulted in significant increment in the antioxidant capacity and polyphenol content of yogurt	Postolache et al. (2023)
	Roselle (*Hibiscus sabdariffa* L.) marmalade	15, and 20%	The marmalade addition enhanced the dry matter, titratable acidity, viscosity, and ash whereas decreased pH, protein, and fat values; marmalade addition increased the antioxidant activities of yogurt samples significantly; the sensory evaluation results showed that samples with 20% marmalade usually received higher scores	Arslaner et al. (2021)
Ice cream	Butterfly pea (*Clitoria ternatea* L.) extract and petals	0.3% (extract), 1, 3, and 5% petals	Sugar-free ice cream prepared with petals and flower extract of butterfly pea demonstrated good potential for development of a healthy dessert product possessing natural colouration, lower glycaemic response, and antioxidative properties	Limsuwan et al. (2014)
Gouda-type cheese	Lavender (*Lavandula angustifolia*) powder	15, 20, 25, and 30 g	Supplementation of milk with flower powder of lavender (0.5 g/L) used in manufacture of Gouda-type cheese did not interfere with oxidative and lipolytic processes significantly	Semeniuc et al. (2022)

TEAC: Trolox equivalent antioxidant capacity

### Milk-based food

Yogurts with *Cucurbita pepo* L. or zucchini flowers displayed higher mineral contents as compared to control samples (El-Sayed et al. 2024). Yogurt with zucchini flowers at 2.5% exhibited the maximum diacetyl content. Addition of zucchini flowers significantly increased total phenols, carotenoids, and antioxidant activity in comparison to the control (Table 4). Cohesiveness, gumminess, chewiness, and hardness increased with an increase in zucchini flower levels, whilst springiness decreased. The sensory evaluation showed that yogurts containing zucchini flowers at 1%, 1.5%, and 2% received higher scores with respect to flavor, appearance, and body & texture than the sample with zucchini flowers at 2.5% (El-Sayed et al. 2024). Yogurt formulated using microencapsulated saffron flower extracts exhibited better functional properties, viz., antioxidant properties and total phenolic content (TPC), which remained stable during a 21-day storage period. Enriched yogurts also displayed a good profile for soluble sugars and organic acids, mainly lactose and lactic acid (Cerdá et al. 2023). Roriz et al. (2018) have developed ice cream by the addition of betacyanin extracted from *Gomphrena globosa* flowers, and their results revealed that the developed ice cream showed considerable colour, nutritional, individual fatty acids, and sugars profiles. The researchers stated that the positive outcomes induced by the incorporation of the natural colourant were preserved throughout storage time.

### Meat-based food

Duggirala and co-workers (2024) have reported that the incorporation of rose (*Rosa canina* L.) and roselle powders into patties containing raw ground beef improved their antioxidant capacity, reduced oxidative markers (carbonyls, Schiff bases, free thiols, 2-thiobarbituric acid reactive substances (TBARS) over storage. In addition, reduced pH and enhanced water-holding capacity were observed amongst all treated patties, having minimal impact on the texture. However, the roselle powder exhibited beneficial effects, but rose powder-treated patties showed superior overall results. Similarly, beef patties prepared from roselle flower showed significantly lower bacterial counts as compared to the control sample (Villasante et al. 2020). A study by Santos et al. (2022) has found that the inclusion of *Cucurbita maxima* or pumpkin flower additives into patty formulation improved sensory parameters of the prepared chicken patties along with consumer acceptance on cold storage. 2% addition of extract from *Moringa* flower (MF) in chicken nuggets not only significantly increased the lightness/decreased the redness but also reduced the hardness, chewiness, and gumminess of the product as compared to control (Madane et al. 2019). Furthermore, incorporation of the flower extract significantly enhanced the odour and oxidative stability scores by decreasing lipid oxidation on storage. Ding et al. (2015) have claimed that the inclusion of polyphenol-rich *Litchi chinensis Sonn*., or litchi flowers (LFs), in emulsified meatballs of pork delayed protein and lipid oxidation. Moreover, owing to the high myofibrillar protein contents present in LF emulsified meatballs, better water-holding capacity and textural profile of meatballs were achieved. In addition, although incorporation of LFs made the emulsified pork meatballs darker and redder, those with LFs at 0.5% exhibited overall best sensory panel acceptance.

### Beverages

Wahyanto and Agustini (2024) have developed a fermented beverage, Kombucha, from *Camellia sinensis* or tea by the addition of *Clitoria ternatea* or butterfly pea flower extract. The study showed that kombucha enrichment with flower extract tends to increase the flavonoid content, a health-beneficial compound, and displays potential anti-inflammatory activity. Incorporation of dandelion (*Taraxacum officinale*) and marigold flowers led to increased polyphenol content in ale beer (Mitreva et al. 2024). Significant increase in the antioxidant activity was also reported for the different variants with flowers (56.6% for dandelion and 54% for marigold). Flowers of *Cannabis sativa* L. were used to extract the essential oil (EO) as well as served as flavouring agents for 2 artisanal alcoholic beverages (a liqueur and a beer) (Ascrizzi et al. 2020). Results showed that the flavour bouquet enrichment was more evident with the liqueur, retaining more compounds derived from hemp. However, the beer sample maintained the volatile aroma compounds profiling, slightly rich in more balsamic notes.

### Edible films/coatings

Edible packaging has lately resurfaced as a viable option for customers due to its capacity to overcome the issues associated with plastic packaging, since it is environment-friendly and biodegradable (Ribeiro et al. 2021). Because it is environmentally benign and biodegradable, edible packaging has recently emerged as a viable choice for consumers, overcoming the problems connected with plastic packaging (Ribeiro et al. 2021). In a recent development, analysis of edible flowers has been carried out for their possible use in improving edible coatings and functional films. This includes extracting cellulose, extracts, and bioactive components from edible flowers (Table 5). The resulting films have structural, mechanical, functional, barrier, and physical qualities because they include bioactive substances that act as natural additives (Asikkutlu and Yildirim 2025; Mary et al. 2024; Saravanan et al. 2024; Jiang et al. 2023; Thokchom et al. 2023; Xie et al. 2023).

**Table 5:** Coating/edible films derived from various edible flower parts with their applications

Common/scientific name	Form used	Quantity added	Matrix used	Food applications	Benefits	References
Butterfly pea/*Clitoria ternatea* L.	Extract	1, 1.5, and 2%	Furcellaran − gelatin	Salmon	Coating with addition of *Clitoria* extract showed an inhibition effect on the growth of microbes throughout the whole storage time for the fishs	Grzebieniarz et al. (2024)
Butterfly pea/*Clitoria ternatea* L.	Anthocyanins	3, 6, and 9%	Starch/Agar	Prawns	The starch/agar/butterfly pea extract (S/A/BPE) films application on freshwater prawns and tiger prawns resulted in changes in colour from dark blue to intense green, i.e., at day 0 (fresh stage) to at day 2 (day for beginning of spoilage), thus indicating the sample spoilage; results indicated that S/A/BPE film possessed the potential for use as a pH based indicator film for detection of freshness in the prawn samples	Saravanan et al. (2024)
Banana/ *Musa paradisiaca* ABB	Nanocellulose	NA	Cellulose	Black grapes	Nanocellulose film wrapped black grapes displayed higher shelf life along with good biodegradability (23%) in comparison to the cellulose film wrapped grapes	Thokchom et al. (2023)
Butterfly pea/*Clitoria ternatea* L.	Anthocyanins	5, 10, 15, 20, and 25%	Gelatin/pectin	Tilapia fish	The film displayed a visible change of color after storing it for seven days at 4 °C, viz., from dark blue to bluish-grey to olive to deep green; the content of total volatile basic nitrogen (TVB-N) changed along with pH change influenced the film’s color response	Narayanan et al. (2023)
Indian azalea/*Rhododendron simsii*	Anthocyanins	2, and 4%	Locust bean gum/polyvinyl alcohol	Shrimp	The films with 2 and 4% of anthocyanins could effectively alter their colors as the total volatile basic nitrogen level exceeded the safe limit in the shrimp, which demonstrated the film suitability for indication of the degree of freshness for the shrimp	Li et al. (2022)
Roselle/*Hibiscus sabdariffa*	Extract	1.5 and 2%	Starch	Potatoes	Shelf life of edible film wrapped potato at room temperature was observed to be four days and that at lower temperatures was six days	Rahmawati et al. (2022)
Cockscomb/*Celosia cristata* L.	Extract	4, 8, and 12%	Locust bean gum/polyvinyl alcohol	Shrimp	The films made from cockscomb flower extract demonstrated obvious color changes, starting from reddish-purple to yellow/brown on shrimp spoilage	Wu et al. (2021)
Saffron/*Crocus sativus* L.	Extract	1, 2, 3, and 4%	Konjac glucomannan	Sliced cucumbers	4% extract concentration was considered the most effective of all treatments in reducing the sliced cucumber microbial content	Hashemi and Jafarpour (2020)

NA: Not applicable

**Fig. 4:**
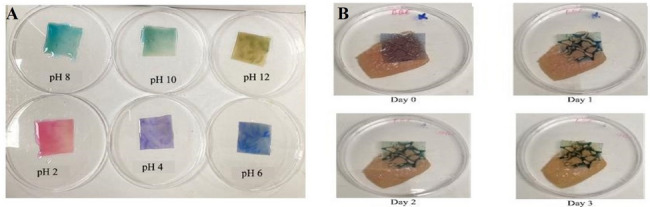
**A** pH indicator films enriched with butterfly pea (*Clitoria ternatea*) flower extract **B** Packaging test of films with chicken breast meat spoilage for 3 days of storage at 25 °C (Asikkutlu and Yildirim 2025; under the CC BY-NC license)

An extract of butterfly pea flower (BBF) was added to the ideal film composition and evaluated for usage as a pH indicator-based smart packaging of chicken breast meat during storage (Fig. 4) (Asikkutlu and Yildirim 2025). On comparison of the properties of the BBF containing film with the optimal film, it was observed that incorporation of the extract resulted in a significant reduction of the tensile strength (TS) along with a significant increment in water solubility, swelling values, and maximal elongation at break (EAB). The films were first affixed to the packages containing chicken breast meat. The pH variations and total mesophilic aerobic bacteria (TMAB) count were then assessed throughout a three-day storage period at room temperature, while the film color changes were also observed. The results revealed a visible color difference in the films on the deterioration of the chicken breast fillet. In another study, various concentrations (1%, 1.5% and 2% w/v) of sugarcane wax (SW) on agar matrix (Agr) in combination with extract of butterfly pea flower (BF) were used to prepare novel pH colorimetric packaging film to monitor the freshness of shrimp (Hashim et al. 2022). The different homogenized sugarcane wax films enhanced the mechanical and physical properties without significant disparity in water vapor permeability and elongation. Remarkably, SW films showed complete protection towards UV–vis light (0%) as well as a significant decrease in visible light. Aydin and Zorlu (2022) found that roselle extract (HE) and alginate films had high antibacterial action against Gram-negative (*Klebsiella pneumoniae* and *Escherichia coli*) and Gram-positive (*Bacillus subtilis* and *Staphylococcus aureus*) bacteria. The films had a stronger antibacterial impact on Gram-positive bacteria, which increased with greater HE concentrations. The resulting films might be used as novel biodegradable and antibacterial films in the food packaging sector to ensure food safety and extend shelf life.

## Overview of the market potential of flowers

Currently, Europe is the leading revenue contributor within this section, remarkably due to the increasing demand for cut flowers in Germany and France, while the Netherlands is projected to remain the largest exporter of cut flowers and bulbs by 2027 (Takahashi et al. 2020). In the Latin America region, Colombia has emerged as the second biggest cut flowers exporter in 2013, exporting 1.35 billion USD worth of carnations, chrysanthemums, roses, among other flowers (Moran 2019). The floriculture market in the Asia-Pacific region has expanded, with the main contribution of India and China (Takahashi et al. 2020). Favorable agroclimatic conditions along with lower labor costs are the key factors responsible for offering an advantage to floricultural products exportation in the Asia-Pacific region (Takahashi et al. 2020). Though the data availability in the edible flowers market is very limited, Table 6 provides some related data. Most of the edible flower processing companies prefer to sell these fresh and untreated. However, some of these companies also process edible flowers to extend their shelf life, which are subsequently sold in the markets in crystallized or dried form (Jadhav et al. 2023). For instance, processed edible flowers are more expensive than the freshly available ones, and edible flowers are sent to the market off-season and will be costlier as compared to the same flower when sold during the harvesting season (Jadhav et al. 2023). The edible flowers market is emerging at a fast rate, owing to the increase in consumer demand. Consequently, to meet this ever-rising demand, producers need to come forward and counter this challenge to grow a greater number of edible flowers to meet the growing demand throughout the world (Jadhav et al. 2023).

**Table 6:** Commercially available diverse edible flowers (Jadhav et al. 2023; under the CC BY-NC-ND license)

Flowers verity	Quantity sold and price	Company
Borage	20 units /501.2 ₹/5.41 €	Innoflower, Spain
Crystallized violas	12 units/ 1747.7 ₹/18.87 €	Meadowsweet flowers, United Kingdom
Cucumber flowers	20 units/ 174.7 ₹/1.89 €	Melita Tarim, Turkey
Crystallized cornflower	12 units/ 1996.1 ₹/215.54 €	Meadowsweet flowers, United Kingdom
Crystallized flower mixture	20 units/ 3193.8 ₹/34.49 €	Meadowsweet flowers, United Kingdom
Crystallized calendula	10 units/ 865 ₹/9.34 €	Farmtapp, Nigeria
Crystallized pansies	10 units/ 865 ₹/9.34 €	Farmtapp, Nigeria
Calendula	6 units/ 218.2 ₹/2.36 €	Kahikatea farm, New Zealand
Dried edible lavender	603.28 ₹/6.51 €	Petite Ingredients, Australia
Dried edible marigold	785.15 ₹/8.48 €	Petite Ingredients, Australia
Dried cornflower petals	594.4 ₹/6.42 €	Meadowsweet flowers, United Kingdom
Edible flower mix	40 units/ 2291.5 ₹/24.74 €	Eu Pantry, Taiwan
Flower mixture	20 units/ 884.5 ₹/9.55 €	Fruttaweb, Italy
Flower mix	25 g/ 78.07 ₹/0.84 €	Gourmet Delight, Mumbai, India
Hibiscus	50 units/ 1009.6 ₹/10.90 €	Gourmet sweet botanicals, California, USA
Lavender	50 units/ 1299.7 ₹/14.04 €	Maddocks farm organics, UK
Pansy	20 units/ 603.2 ₹/6.51 €	Flores Frescas, Spain
Pineapple sage	40 units/ 709.7 ₹/7.66 €	Innoflower, Spain
Roses	10 units/ 643.2 ₹/6.95 €	Flores Frescas, Spain
Violas	60 units/ 998.08 ₹/10.78 €	Maddocks farm organics, UK
Yunnan rose petals	1000 g/ 855.2 ₹/9.24 €	Eu Pantry, Taiwan

Prices are mentioned in Indian currency and Euro

## Safety issues of consuming edible flowers

Edible flowers have been enjoyed for consumption since ancient times. Our ancestors distinguished between both edible and non-edible flowers based on the presence of certain chemicals and alkaloids, which are part of the plants’ natural defense systems (Chetia et al. 2025). They recognized edible flowers as those containing beneficial alkaloids that also offer pharmacological activities, primarily from medicinal plants (Chetia et al. 2025). While some flowers contain alkaloids that can be toxic and have stimulant or psychotropic effects, these are typically considered non-edible (Nicolau and Gostin 2016). Other factors, such as the introduction of microbiological hazards (*Salmonella* spp.), biological hazards, and chemical hazards (dimethoate (insecticide), sulphites, and diethyl-meta-toluamide (insect repellent) to edible flowers, can render these flowers non-edible (Matyjaszczyk and Śmiechowska 2019; Nicolau and Gostin 2016). Even though edible flowers may contain some anti-nutrients, Khan and colleagues (2017) found that an oral dosage of up to 6000 mg/kg using an aqueous extract of *Butea monosperma* or Flame of the Forest was safe, depending on the concentration and permissible limits. The traditional extract of whole *Musa balbisiana*, or bhimkol flower, included phytate, tannin, alkaloids, and cyanogenic glycoside at 46.90 mg phytic acid/100 g, 14.3 mg tannic acid equivalent (TAE)/100 g, 2.78 mg/100 g, and 0.0001%, respectively (Muchahary and Deka 2021). The anti-nutritional substances found in the flowers of *Carica papaya* (papaya), *Cucurbita maxima* (pumpkin), and *Allium cepa* (onion) include tannin, oxalate, alkaloids, saponin, and phytate, as measured by Halder and Khaled (2021) per 100 g of fresh flowers. The tannin content of *Allium cepa* (onion), *Cucurbita maxima* (pumpkin), and *Carica papaya* (papaya) was found to be 0.44, 2.16, and 1.72 mg/100 g, respectively. Moreover, phytate, alkaloids, saponin, and oxalate were detected in *Allium cepa* (onion) at 3.06, 0.88, 850, and 1.52 mg/100 g; those in *Carica papaya* (papaya) at 6.58, 0.18, 230, and 3.18 mg/100 g; and lastly in *Cucurbita maxima* (pumpkin) at 5.07, 0.35, 50, and 0.2 mg/100 g respectively. According to research by Aung et al. (2020), phytates (mg/100 g) were detected in the blooms of blue water lilies, Chinese water lilies, and white-water lilies at 304, 304, and 456, respectively. The increased fiber content in them may be the cause of this higher content. These edible flowers may potentially contain certain anti-nutrients. To safely include edible flowers into everyday meals while avoiding potential risks and optimizing the nutritional advantages, it is necessary to know the anti-nutrient content in edible flowers and their dietary consumption (Chetia et al. 2025).

## Strengths and shortcomings

The current review is remarkably novel and comprehensive in approach, effectively consolidating a wide range of applications (synthesis of metallic nanoparticles and green carbon dots, biochar, bio-adsorbents, functional food developments, and essential oils production) wherein the edible flowers have been employed efficiently. The majority of reviews published during recent years have mainly focused on food and health applications of the edible flowers. Nonetheless, the review offers its limitations. For instance, numerous non-edible flowers have constantly been used for synthesizing green carbon dots, biochar, bio-adsorbent, and metallic nanoparticles. This is the only reason for using the word “flowers” title instead of the word “edible flowers” in our study, but the valorization of non-edible flowers equally contributes towards achieving the SDGs, such as better nutrition, ensuring hygienic living conditions, clean water and sanitation, industry and profitable jobs, and constructing robust infrastructure. In addition, several edible flower-derived food items are commercially marketed. However, the true commercial potential of such items in terms of real-world pricing, customer demand, and production costs has not been adequately addressed or investigated in prior research.

## Conclusion

Flowers valorization as green biomass to value-added products has proven their potential in various sectors such as food, health, environment and among others, which fits with the circular bioeconomy concept and various SDGs. For synthesizing CDs, MNPs, bio-adsorbents, and biochar’s, the researchers have explored a variety of flowers that directly help in improving the environment, particularly purifying water and wastewater, concrete’s compressive strength, human health especially targeting harmful bacteria, fungus, and cancer cell lines. While for various food applications, the edible flowers play a crucial role in the design of different kinds of functional foods with improved nutritional quality. Although the flowers possess many anti-nutritional factors yet their safe employment in diverse food enrichment applications has been proven in numerous pre-clinical studies (Książkiewicz et al. 2025; Núñez et al. 2023; Lv et al. 2020).

## Data Availability

Data will be made available on request.
